# Factors associated with discordance between pathological and radiological tumor size and risk of re-excision in breast-conserving surgery: a post-hoc analysis within a randomized trial

**DOI:** 10.1007/s10549-026-08052-x

**Published:** 2026-08-13

**Authors:** Christos Kollatos, Eirini Pantiora, Allan Jazrawi, Fredrik Wärnberg, Staffan Eriksson, Andreas Karakatsanis

**Affiliations:** 1 Department of Surgical Sciences, Uppsala University, Akademiska Sjukhusvägen, Ingång 70, Uppsala, 751 85 Sweden; 2 Section for Breast Surgery-Department of Surgery, Uppsala University Hospital, Uppsala, Sweden; 3 Centre for Clinical Research Västmanland, Uppsala University, Västerås, Sweden; 4 Section for Breast Surgery-Department of Surgery, Västmanlands County Hospital, Västerås, Sweden; 5 Department of Reconstructive Plastic Surgery, Karolinska University Hospital, Stockholm, Sweden; 6 Region Västra Götaland, Department of Surgery, Sahlgrenska University Hospital, Gothenburg, Sweden; 7 Department of Surgery, Institute of Clinical Sciences, Sahlgrenska Academy, University of Gothenburg, Gothenburg, Sweden

**Keywords:** Breast, Breast cancer, Breast-conserving surgery, Magnetic resonance imaging, Reoperation, Treatment outcome

## Abstract

**Purpose:**

Accurate preoperative radiological assessment is essential in breast-conserving surgery (BCS) to achieve clear margins while minimizing unnecessary healthy tissue excision. Discrepancies between radiological and pathological tumor size may contribute to re-excision. This study evaluated factors associated with radiology–pathology discordance and their impact on re-excision.

**Methods:**

This post hoc analysis of the MagTotal randomized trial included patients with non-palpable breast cancer undergoing BCS with lesion localization and sentinel lymph node dissection. The Pathology-to-Radiology Ratio (PRR) quantified discordance between size on preoperative imaging and specimen pathology. Primary outcomes were predictors of PRR and re-excision. A multivariable model was used to construct a nomogram for predicting PRR.

**Results:**

Among 414 patients (median age 65 years; median tumor size 10.3 mm), MRI was performed in 24.4%. Median PRR was 1.25, suggesting systematic underestimation. MRI consistently overestimated tumor size in most subtypes but provided more accurate estimates for invasive lobular carcinoma (ILC), compared with conventional imaging. Independent predictors of discordance included ILC, high grade, and small tumor size. Eleven patients (2.7%) required re-excision, which was associated with tumor biology and mismatches between imaging and pathology but not with MRI use.

**Conclusion:**

Re-excision in non-palpable breast cancer is primarily driven by tumor biology and radiology–pathology mismatch. Although MRI improved size assessment in ILC, it did not reduce re-excision rates. Integrating imaging with biological factors, supported by predictive tools such as a nomogram, may refine surgical planning in BCS.

## Introduction

Accurate preoperative tumor size assessment is essential in breast-conserving surgery (BCS) to achieve clear margins while minimizing unnecessary removal of healthy tissue. This is particularly important in cases of non-palpable breast lesions, where preoperative localization is required. Inaccurate measurements may result in inadequate margin control and the need for re-excision, or in excessive removal of healthy surrounding tissue, which increases the risk of complications and suboptimal cosmetic outcomes [1].

Magnetic resonance imaging (MRI) has emerged as a valuable tool in preoperative planning because of its high sensitivity (> 90%) for detecting multifocal, multicentric, or contralateral disease [2, 3], particularly in dense breast tissue [4]. However, its variable specificity (30–90%) raises concerns regarding false positives, overdiagnosis, and overtreatment [5–8]. Moreover, MRI has not demonstrated a consistent impact on re-excision rates, even when assessing challenging tumor types such as invasive lobular carcinoma (ILC) or ductal carcinoma in situ (DCIS) [9–11]. Several factors—including non-palpable lesions, the presence of non-mass-like enhancement at magnetic resonance imaging, multifocality, the presence of a DCIS component, and pre- and post-operative size discordance—have been shown to be significantly associated with re-excision [12, 13]. Breast density may also influence the accuracy of lesion extent estimation [14, 15]. Most of these findings, however, derive from retrospective studies lacking standardized patient selection or data collection, introducing potential bias and requiring cautious interpretation.

The recent Magtotal (Magnetic Marker to Detect Primary Lesion and Sentinel Node in Breast Cancer) phase 3, open-label, pragmatic RCT demonstrated equivalence between a ferromagnetic seed and guidewire for lesion localization in both re-excision rates and resection ratios, defined as the ratio of excised volume to optimal resection volume (ORV: tumor volume plus 1-cm margins, as calculated by preoperative radiology) [16]. The study population included patients undergoing BCS with preoperative lesion localization and magnetic-guided sentinel lymph node dissection (SLND). As patients requiring mastectomy and those with palpable lesions were excluded, the dataset was well suited for this analysis.

## Methods

The trial has been presented elsewhere in detail [16]. In brief, the study was conducted between May 2018 and May 2022 in three Swedish hospitals: Akademiska University Hospital (Uppsala), Västmanlands Hospital (Västerås), and Sahlgrenska University Hospital (Gothenburg). Eligible patients had non-palpable DCIS or T1–T3 invasive breast cancer and were scheduled for BCS and SLND. In addition to the exclusion criteria defined in the primary trial, patients who had received primary systemic therapy or whose tumors were identified solely by MRI were also excluded from this analysis. The trial was registered in a public trial registry (ISRCTN 11914537, registration date 01/05/2018).

All available radiologic modalities were reviewed during surgical planning and the tumor size guiding excision extent was defined by the clinically most relevant modality. For solid lesions, ultrasound (US) was the primary modality, whereas mammographic extent was used for microcalcifications. Preoperative MRI was not routinely performed but was considered in cases of discrepancy. The ORV was subsequently calculated based on the modality depicting larger tumor size, to ensure negative margins and its documentation was a mandatory part of surgical planning.

Preoperative radiological accuracy was quantified by reviewing preoperative radiology and postoperative specimen reports and defining the ratio between the two (Pathology-to-Radiology Ratio; PRR). A ratio > 1 denoted extent underestimation in preoperative imaging (and thus a potentially higher risk of re-excision), whereas a ratio < 1 indicated overestimation (and potentially removal of larger volumes than necessary).

The primary outcome was factors associated with re-excision. Negative margins were defined as “no tumor on ink” for invasive cancer and ≥ 2 mm for DCIS. Associations between PRR and different factors were also examined.

### Statistical analysis

Categorical variables, presented as frequencies and percentages, were analyzed using the Chi-square test or Fisher’s exact test. Continuous variables were summarized as medians with interquartile ranges (IQR). Paired continuous variables were compared using the Wilcoxon signed-rank test. Correlations were assessed with Spearman’s rho, while agreement between repeated patient measurements was evaluated using the intraclass correlation coefficient (ICC). The Bland-Altman method was used to assess systematic bias in preoperative assessment by plotting the difference between PRR values against their mean.

Univariable analyses were first conducted to explore crude associations between each variable and the outcomes of interest. Logistic regression was used for binary outcomes, and linear or generalized linear models were applied to continuous outcomes according to their distribution. Because this was a post hoc analysis, variables included in the multivariable models were selected based on clinical relevance and prior inclusion in the primary trial analysis, rather than solely on statistical significance in univariable testing. To account for the limited number of events and reduce the risk of overfitting, inferential Least Absolute Shrinkage and Selection Operator (LASSO) regression was also applied, incorporating clinically important variables regardless of univariable significance [17]. Effect estimates were reported with 95% confidence intervals (CIs), and all models were assessed for multicollinearity. A nomogram was constructed as a prediction model for PRR as a continuous outcome using multivariable linear regression based on the statistically significant variables from the previous LASSO regression model. The model was fitted using ordinary least squares linear regression and was used to construct the nomogram via the rms package [18].

All analyses were conducted using Stata Statistical Software: Release 17 (College Station, TX, USA: StataCorp LLC) and R 4.4.0 (R Core Team, 2024). The study adhered to the STROBE (Strengthening the Reporting of Observational Studies in Epidemiology) guidelines [19].

## Results

The initial trial included 426 patients. Seven patients had received primary systemic therapy, and in five patients the tumor was identified solely by MRI. This resulted in a final analysis of 414 patients (median [IQR] age: 65 [56, 71] years; Body Mass Index: median [IQR] 26.6 [24.0, 29.8] kg/m²; imaging lesion size: median [IQR] 10.3 [8, 15] mm; Table 1). Of these, 340 (82.1%) had invasive ductal carcinoma (IDC), non-special type (NST); 42 had ILC (10.1%); 26 had special types of invasive carcinoma such as mucinous, medullary, or apocrine (6.3%); and six had DCIS with suspicion for micro-invasion (1.5%). Multifocality was present in 13 patients (3.1%).

**Table 1:** Basic patient and tumor characteristics (*N* = 414)

Characteristic	Results
Age, median (IQR), years	65 (56–71)
BMI, median (IQR), kg/m²	26.6 (24.0–29.8)
Imaging lesion size, median (IQR), mm	10.3 (8–15)
Histological subtype, n (%)	
– IDC (NST)	340 (82.1%)
– ILC	42 (10.1%)
– DCIS	6 (1.5%)
– Other^a^	26 (6.3%)
Multifocality, n (%)	13 (3.1%)
Preoperative MRI performed, n (%)	101 (24.4%)
– Västerås, n (%)	77 of 106 (73.0%)
– Uppsala, n (%)	24 of 232 (10.3%)
– Gothenburg, n (%)	0 of 76 (0.0%)
Preoperative suspicion of multifocality, n (%)	
– Yes	7 of 13 (53.9%)
– No	94 of 401 (23.4%)
Surgical procedure, n (%)	
– WLE	346 (83.6%)
– OPBCS	49 (11.8%)
– Therapeutic mammaplasty	19 (4.6%)
Minimum margin, median (IQR), mm	
– Intraoperative specimen radiology	9.8 (6–14)
– Definitive specimen pathology	5.0 (3–9)
PRR, median (IQR, range)	1.25 (1–1.75; range 0.24–7.17)
Re-excision performed, n (%)	11 (2.7%)

Abbreviations: IQR, Interquartile Range; BMI, Body Mass Index (kg/m^2^); MRI, magnetic resonance imaging; IDC (NST), invasive ductal cancer (nonspecific type); ILC, invasive lobular cancer; DCIS, Ductal cancer in situ; WLE, wide local excision; OPBCS, oncoplastic breast-conserving surgery; PRR, pathology-to-radiology ratio. ^a^Other refers to mucinous, medullary, and tubular breast cancers

Preoperative MRI was performed in 101 patients (24.4%). Exploratory analyses suggested that MRI use was predominantly associated with trial site (Västerås: 77 of 106 [73.0%]; Uppsala: 24 of 232 [10.3%]; Gothenburg 0 of 76 [0.0%]; *p* < .001) and with suspicion of multifocal/multicentric disease on conventional imaging (seven of thirteen [53.9%] vs. 94 of 401 [23.4%], *p* = .018).

Wide local excision with tissue rearrangement was performed in 346 patients (83.6%), while 49 (11.8%) underwent oncoplastic breast conservation and 19 (4.6%) a therapeutic mammaplasty. The median minimum margin on intraoperative specimen radiology was significantly larger than on definitive specimen pathology (9.8 mm [IQR 6–14] vs. 5 mm [IQR 3–9], *p* < .001). The median PRR was 1.25 (IQR 1–1.75; range 0.24–7.17). Eleven patients (2.7%) underwent re-excision due to positive margins, which is slightly different from the previously reported re-excision rate (2.9%) in the full trial population of 426 patients.

## PRR of imaging modalities

A moderate correlation was observed between the PRR of MRI and conventional imaging (rho = 0.61, 95% CI 0.46–0.761, *p* < .001; ICC = 0.927, 95% CI 0.676–0.999). Linear regression further supported this finding (beta 0.392, 95% CI 0.314–0.471, *p* < .001).

**Fig. 1:**
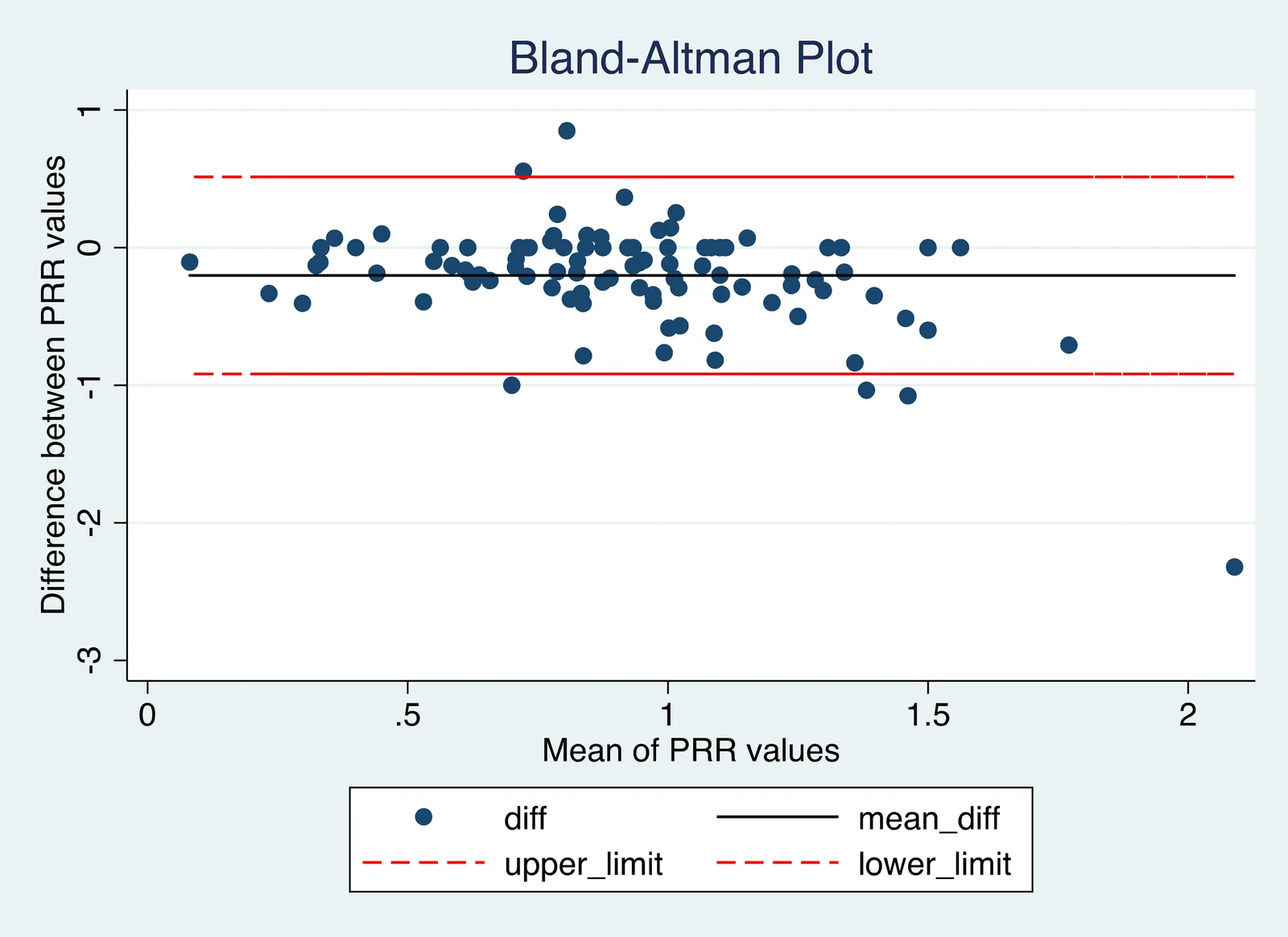
Bland–Altman plot showing the differences between the pathology-to-radiology ratio (PRR) of magnetic resonance imaging (MRI) minus conventional radiology and the mean PRR of these modalities. The red lines indicate the 95% limits of agreement

**Fig. 2:**
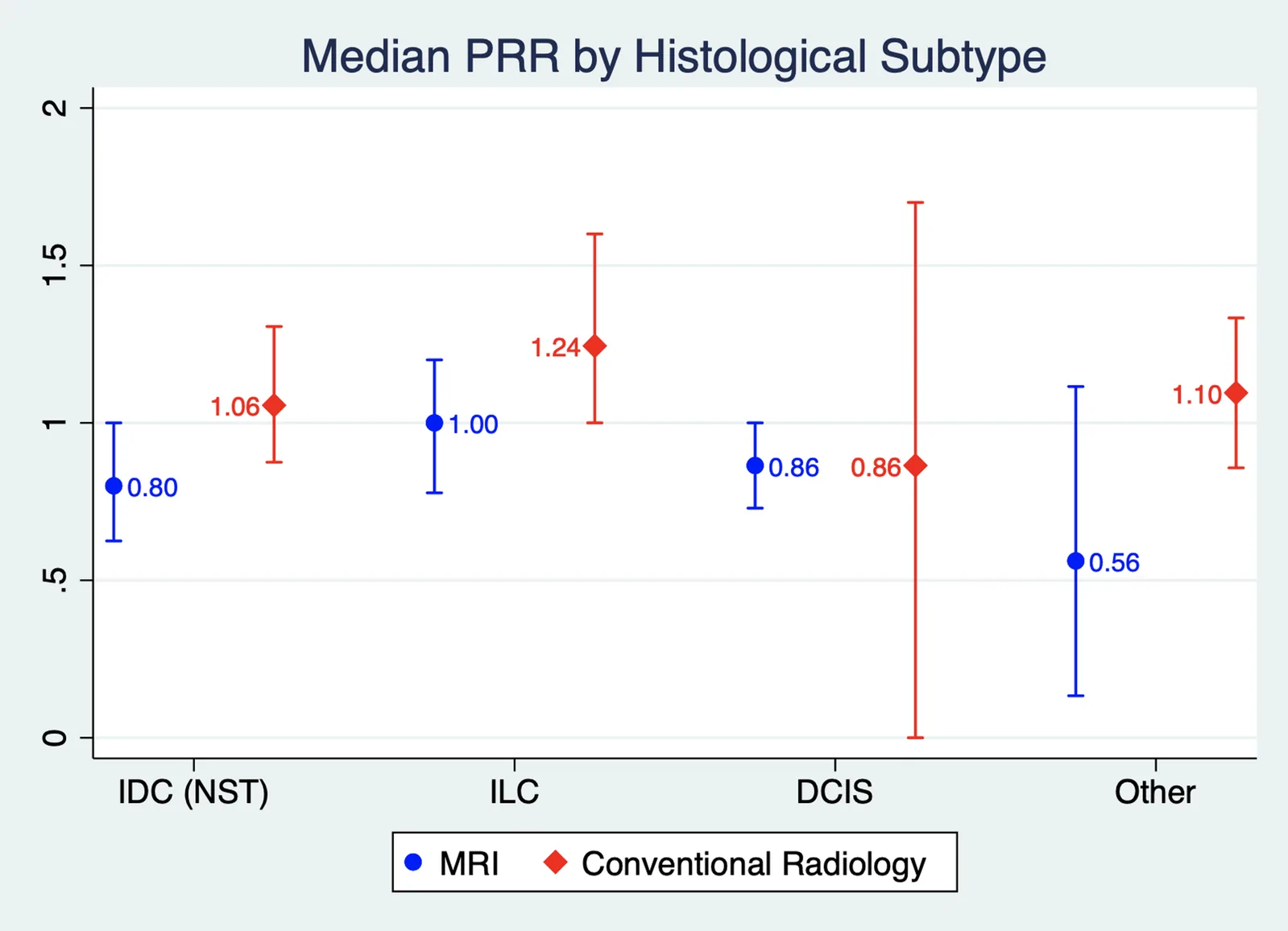
Median pathologic-to-radiologic ratios (PRR) with interquartile ranges by histological subtype and imaging modality

As shown in Fig. 1, MRI consistently overestimated tumor size, with a mean PRR difference of -0.2 and 95% limits of agreement from − 0.92 to 0.52. Twelve cases were identified as outliers. Among these, six had IDC (NST), two had ILC, and four had other cancer types. Two patients had multifocal or multicentric disease. None required re-excision. PRR differed across histological subtypes and imaging modalities, with median values for MRI (blue) and conventional radiology (red) shown in Fig. 2.

## Association analyses for PRR

The results of the univariable and multivariable analyses examining factors associated with PRR are presented in Table 2.

**Table 2:** Univariable and multivariable analysis for the pathology-to-radiology ratio (PRR)

Variable	Univariable Analysis		Multivariable Analysis	
	Coefficient (95% CI)	*p* value	Coefficient (95% CI)	*p* value
Age	0.023 (− 0.009 to 0.014)	0.715	−0.001 (− 0.007 to 0.004)	0.668
BMI	−0.004 (− 0.221 to 0.014)	0.664	0.000 (− 0.010 to 0.011)	0.952
Tumor size	−0.255 (− 0.035 to 0.155)	< 0.001	−0.026 (− 0.035 to − 0.018)	< 0.001
Preoperative MRI	1.14 (0.90 to 1.71)	0.020	−0.017 (− 0.171 to 0.137)	0.829
Multifocal disease	0.265 (− 0.678 to 1.191)	0.590	0.564 (0.115 to 1.014)	0.014
**Histology**				
IDC (NST)	Reference		Reference	
ILC	0.503 (0.054 to 0.952)	0.028	0.288 (0.063 to 0.514)	0.012
DCIS	0.348 (0.096 to 0.600)	0.007	−0.020 (− 0.334 to 0.294)	0.900
Other^a^	0.427 (− 0.205 to 1.058)	0.185	0.189 (− 0.175 to 0.554)	0.309
**Grade**				
Grade 1	Reference		Reference	
Grade 2	0.286 (0.084 to 0.488)	0.006	0.188 (0.043 to 0.332)	0.011
Grade 3	0.287 (− 0.023 to 0.552)	0.030	0.297 (0.080 to 0.514)	0.007
**Intrinsic subtype**				
Luminal A	Reference		Reference	
Luminal B, ERBB2 negative	0.027 (− 0.196 to 0.251)	0.810	0.024 (− 0.130 to 0.179)	0.757
Luminal B, ERBB2 enriched	−0.004 (− 0.508 to 0.500)	0.987	−0.181 (− 0.608 to 0.247)	0.408
Basal-like, ERBB2 enriched	0.544 (− 0.309 to 1.399)	0.211	0.091 (− 0.364 to 0.547)	0.694
Triple Negative Breast Cancer	0.125 (− 0.471 to 0.721)	0.679	0.066 (− 0.254 to 0.387)	0.684

*Multivariable analysis adjusted for all variables listed. Abbreviations: BMI, Body Mass Index (kg/m^2^); MRI, magnetic resonance imaging; IDC (NST), invasive ductal cancer (nonspecific type); ILC, invasive lobular cancer; DCIS, Ductal cancer in situ. ^a^Other refers to mucinous, medullary, and tubular breast cancers

Factors significantly associated with PRR in both univariable and multivariable analyses included index tumor size, nuclear grade, and histological subtype. In both models, smaller tumor size was associated with higher PRR (univariable: β = −0.255, 95% CI: −0.035 to 0.155, *p* < .001; multivariable: β = −0.026, 95% CI: −0.035 to − 0.018, *p* < .001). Higher PRR values were observed for grade 2 (univariable: β = 0.286, 95% CI: 0.084–0.488, *p* = .006; multivariable: β = 0.188, 95% CI: 0.043–0.332, *p* = .011) and grade 3 tumors (univariable: β = 0.287, 95% CI: −0.023–0.552, *p* = .030; multivariable: β = 0.297, 95% CI: 0.080–0.514, *p* = .007) compared with grade 1. ILC was associated with higher PRR relative to IDC (NST) in both univariable (β = 0.503, 95% CI: 0.054–0.952, *p* = .028) and multivariable analyses (β = 0.288, 95% CI: 0.063–0.514, *p* = .012).

Several factors were significantly associated with PRR in univariable analysis only. Preoperative MRI use was associated with lower PRR (β = 1.14, 95% CI: 0.90–1.71, *p* = .020), and DCIS was associated with higher PRR compared with IDC (NST) (β = 0.348, 95% CI: 0.096–0.600, *p* = .007); however, these associations were not retained in multivariable analysis. Multifocal or multicentric disease was not significant in univariable analysis (β = 0.265, 95% CI: −0.678 to 1.191, *p* = .590) but was independently associated with higher PRR in multivariable analysis (β = 0.564, 95% CI: 0.115–1.014, *p* = .014).

All remaining variables, including age, BMI, other histological subtypes, and intrinsic molecular subtype, were not significantly associated with PRR in either univariable or multivariable analyses.

### Sensitivity analysis

Adaptive double-selection Lasso regression, incorporating the same predictors and adjusted for age and intrinsic molecular subtype as clinically relevant controls, identified associations between PRR and ILC (β = 0.49, 95% CI: 0.04–0.95, *p* = .035); grade 2 (β = 0.29, 95% CI: 0.06–0.51, *p* = .012); and grade 3 tumors (β = 0.40, 95% CI: 0.07–0.74, *p* = .019). Tumor size was inversely associated with PRR (β = −0.04, 95% CI: −0.06 to − 0.02, *p* < .001). Multifocality was not statistically associated with PRR (β = 1.01, 95% CI: −0.04 to 2.07, *p* = .060).

DCIS (β = 0.03, 95% CI: −0.52–0.58, *p* = .927), other histological subtypes (β = 0.35, 95% CI: −0.35–1.05, *p* = .326), and MRI status (β = −0.04, 95% CI: −0.30–0.22, *p* = .791) were also not significantly associated with PRR. Additional covariates, including BMI, recruitment site, screening status, and lesion palpability, were considered but not selected by Lasso.

**Fig. 3:**
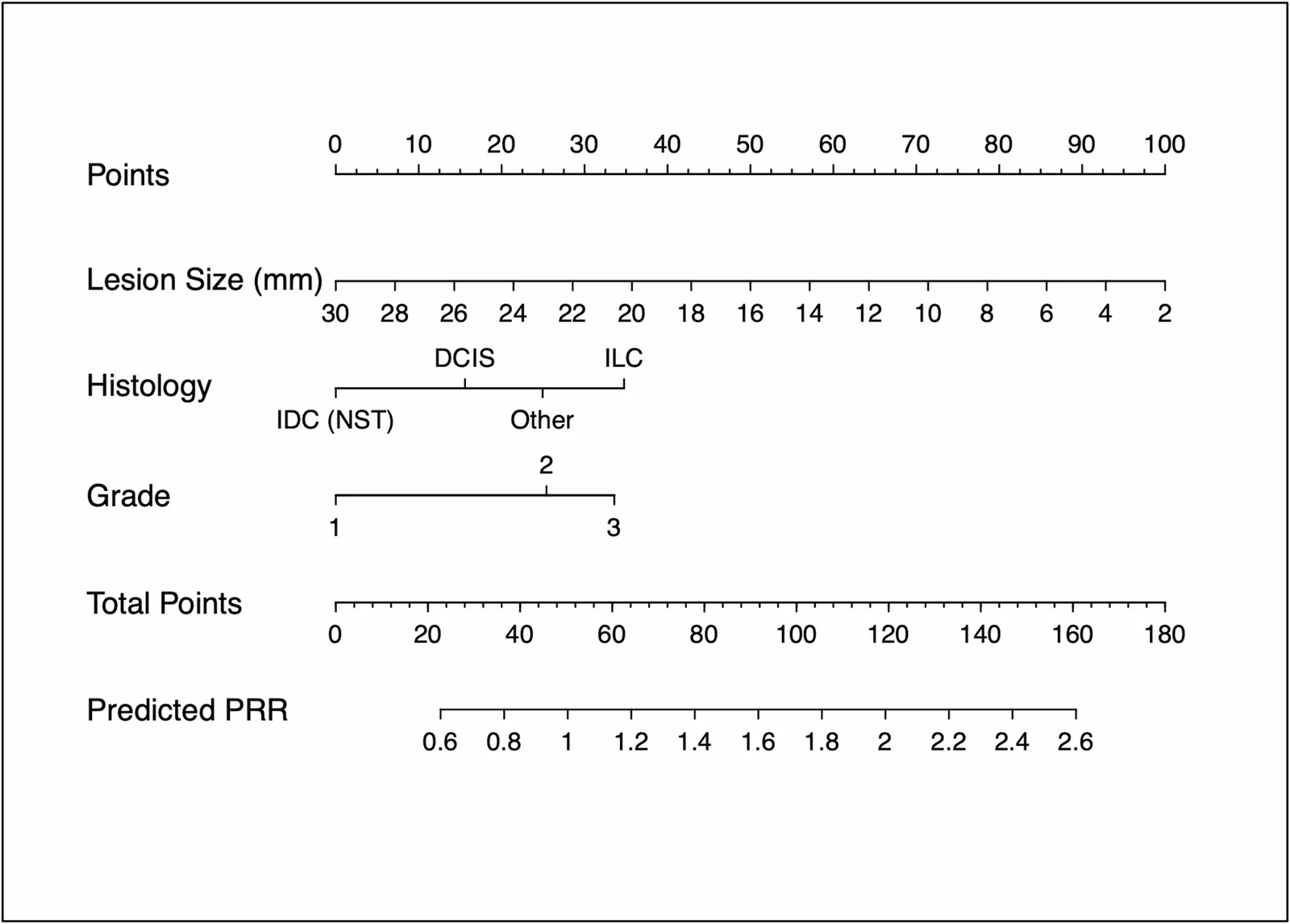
Nomogram for predicting pathology-to-radiology ratio (PRR) in breast tumors

A nomogram was developed integrating lesion size, histological subtype, and tumor grade to predict PRR (Fig. 3). Among the patients, 400 (96.6%) had tumor lesions measuring up to 30 mm, which is why 30 mm was chosen as the upper limit.

## Association analyses for re-excision rates

The results of the univariable and multivariable analyses examining factors associated with re-excision are presented in Table 3.

**Table 3:** Univariable and multivariable analysis for re-excision

Variable	Univariable analysis		Multivariate analysis	
	Coefficient (95% CI)	*p* value	Coefficient (95% CI)	*p* value
**Site**				
Uppsala	Reference	—	—	—
Västerås	−1.51 (−3.22 to 0.21)	.085	−0.054 (−0.124 to 0.016)	.133
Gothenburg	−2.09 (−3.75 to −0.43)	.014	−0.047 (−0.106 to 0.012)	.122
**Histology**				
IDC/NST	Reference	—	—	—
ILC	−0.87 (−2.47 to 0.74)	.291	−0.029 (−0.109 to 0.051)	.473
DCIS	−2.25 (−4.53 to 0.02)	.052	−0.303 (−0.915 to 0.308)	.331
Other	−0.64 (−2.78 to 1.49)	.555	−0.022 (−0.115 to 0.070)	.635
**Minimum radiologic margin (mm)**	0.19 (0.04 to 0.34)	.012	0.003 (−0.001 to 0.007)	.097
**PRR**	−0.50 (−0.81 to −0.20)	.001	−0.022 (−0.059 to 0.015)	.246
**Multifocality/Multicentricity**	omitted due to perfect prediction	—	0.032 (−0.013 to 0.076)	.160
**Localisation technique**				
Guidewire	Reference	—	—	—
Magnetic marker	0.21 (−1.00 to 1.41)	.736	0.009 (−0.027 to 0.044)	.632
**Preoperative MRI**	−0.15 (−1.50 to 1.19)	.822	0.016 (−0.043 to 0.074)	.604
**Surgery**				
WLE	Reference	—	—	—
OPBCS	0.36 (−1.72 to 2.44)	.737	−0.011 (−0.057 to 0.035)	.639
Therapeutic mammaplasty	empty	—	−0.023 (−0.082 to 0.036)	.441
**Volume removed to ORV ratio**	−0.02 (−0.08 to 0.03)	.416	0.001 (−0.002 to 0.003)	.565

Abbreviations: IDC (NST), invasive ductal cancer (nonspecific type); ILC, invasive lobular cancer; DCIS, Ductal cancer in situ; PRR, pathology-to-radiology ratio; MRI, magnetic resonance imaging; WLE, wide local excision; OPBCS, oncoplastic breast-conserving surgery; ORV, optimal resection volume. aOther refers to mucinous, medullary, and tubular breast cancers

No variables were significantly associated with re-excision in both univariable and multivariable analyses. Several factors were significantly associated with re-excision in univariable analysis only. Treatment in Gothenburg was associated with lower re-excision rates compared with Uppsala (β = −2.09, *p* = .014), whereas the association for Västerås did not reach statistical significance (β = −1.51, *p* = .085). A larger minimum radiologic margin was associated with re-excision (β = 0.19 per mm, *p* = .012), and higher PRR was associated with lower re-excision rates (β = −0.50, *p* = .001). In multivariable analysis, no variables were significantly associated with re-excision.

### Sensitivity analysis

In the adaptive LASSO model, a higher PRR, denoting more discrepancy was associated with increased odds of re-excision (OR = 2.00, 95% CI: 1.20–3.35, *p* = .008), while a larger minimum radiologic margin remained protective (OR = 0.81, 95% CI: 0.68–0.95, *p* = .013). On the other hand, DCIS was associated with higher odds of re-excision (OR = 12.78, 95% CI: 1.36–119.99, *p* = .026), whereas neither ILC (OR = 4.40, *p* = .078), nor study site [Gothenburg (OR = 19.47, *p* = .055), and Västerås (OR = 15.44, *p* = .063)], reached significance. Other histological subtypes and control variables, including screening status, lesion palpability, age, BMI, multifocality, and luminal subtype, were not retained in the model.

## Discussion

This post-hoc analysis of a RCT demonstrates that re-excision rates in non-palpable breast cancer are primarily influenced by discrepancies between preoperative imaging and final pathological size. Interestingly, preoperative MRI neither improved measurement accuracy nor reduced re-excision rates, whereas ILC histology, smaller tumor size on preoperative imaging, and higher tumor grade were associated with greater discordance between preoperative extent on radiology against definitive postoperative pathology.

The need for re-excision undermines the benefits of breast-conserving surgery by increasing complications, delaying adjuvant therapy, worsening cosmetic outcomes, raising costs, and sometimes driving conversion to mastectomy [20–24]. Previously identified risk factors include younger age, non-palpable tumors, neoadjuvant therapy, microcalcifications, multifocality, lobular histology, hormone status, DCIS, and resection volume [12, 25–27]. Intraoperative margin assessment techniques such as intraoperative radiography, ultrasound, frozen section or imprint cytology and cavity shave have shown a potential to reduce reoperations [28–31]. On the other hand, preoperative MRI, though often implemented, remains controversial [32–36].

Accurate definition of the surgical “target volume” is critical in breast-conserving surgery: although larger resections may reduce margin positivity, they may also increase operative time and risk for complications, making precise assessment particularly important [37, 38]. In this study, tumor characteristics, including histological subtype, grade, and size, were independent predictors of discordance. On the other hand, imaging underestimated tumor extent by approximately 29% for ILC, 19% for grade 2 tumors, and 30% for grade 3 tumors, whereas larger tumors were more probable to be overestimated. These discrepancies have direct implications for preoperative planning and intraoperative decision-making when defining the target volume and suggest that tumor characteristics should be routinely integrated in the preoperative planning.

In the present study, MRI consistently overestimated tumor size in most histological subtypes, except for ILC, in which mammography and ultrasound were less accurate, probably because of diffuse growth patterns and limited microcalcifications [39]. Twelve cases fell outside these 95% limits of agreement, meaning the difference between the MRI-based and conventional-imaging-based PRR for these patients exceeded the range expected from typical measurement variability between the two modalities, rather than reflecting an extreme PRR value in itself. Evidence on routine preoperative MRI is mixed: RCTs show higher mastectomy rates without consistent reductions in reoperations [40–44]; observational studies suggest a benefit in ILC [9, 10], but not in DCIS [11]. While several guidelines have recommended MRI for ILC when BCS is planned, it seems that its use is to primarily investigate multicentricity and bilaterality [45, 46]. At the same time, increased costs, treatment delays, and anxiety from incidental findings need to be factored in when preoperative MRI is considered [47]. In the future, MRI could be utilized to guide treatment de-escalation in low-risk ILC, as suggested by the PROSPECT trial, though its approach relies more on grade and receptor status than size alone [48].

An interesting finding of the study was that a larger minimum intraoperative margin measured on specimen radiography was associated with less risk of re-excision. Although expected, this finding is clinically relevant and suggests that, because the actual-to-calculated resection ratio was not associated with re-excision, securing an adequate margin after excision of the intended volume is crucial. While the MagTotal trial used two-dimensional specimen radiography, emerging real-time margin assessment tools and artificial intelligence–based systems may further improve precision [49–54].

The calculated ORV, defined as the imaging-based tumor volume plus a standardized 1-cm margin, provides a reproducible, protocol-driven target for surgical planning and enables prospective, objective comparison of intended versus achieved resection across surgeons and sites. However, our finding that the ratio of volume actually removed to the calculated ORV was not associated with re-excision, whereas the achieved minimum intraoperative margin was protective, suggests that ORV functions best as a planning benchmark rather than a guarantee of adequate margins. Because ORV is derived directly from preoperative imaging, any underlying imaging inaccuracy, such as that observed in ILC or high-grade tumors, is propagated into the ORV calculation itself. This underscores the rationale for supplementing ORV-based planning with biologically informed correction factors, such as those provided by the nomogram, rather than relying on ORV or resection ratio in isolation. Real-time intraoperative margin assessment tools may further help bridge the gap between a preoperatively defined target volume and the margin ultimately achieved.

In clinical practice, the nomogram could be applied at the time of preoperative planning by entering the imaging-based lesion size together with histological subtype and tumor grade, obtained via core needle biopsy, to generate a predicted PRR. A predicted PRR substantially greater than 1, as seen with ILC, higher-grade tumors, or smaller lesions, would flag a case at higher risk of imaging underestimation, prompting the surgeon to consider a wider excision margin beyond the standard 1-cm margin to reduce the likelihood of re-excision. Conversely, a predicted PRR at or below 1, more likely with larger, lower-grade IDC (NST) lesions, could support a more conservative margin, minimizing unnecessary excision of healthy tissue and improving cosmetic outcome. Used this way, the nomogram would function as a preoperative decision-support tool that personalizes the excision margin according to tumor biology rather than applying a uniform fixed margin to all patients, complementing rather than replacing standard margin protocols. Prospective validation would be required before this approach could be adopted into routine practice.

## Strengths and limitations

Although exploratory, this analysis is based on a well-conducted multicenter pragmatic randomized controlled trial with prospective data collection, minimal missing data, and appropriate statistical methods to reduce bias, including prospectively defined volume calculations. The study population was limited to patients with small lesions eligible for breast-conserving surgery, ensuring baseline comparability and reducing heterogeneity related to tumor size or locally advanced disease. The developed nomogram illustrates the potential value of integrating tumor biology and grade with radiological estimates to refine surgical planning. Re-excision rates were low, supporting the reliability of pathological tumor size assessment. Whilst this resulted to a low number of events, this was addressed by implementing LASSO regression as a prediction-oriented method. Nonetheless, with only 11 re-excision events, all effect estimates, including those reaching statistical significance, should be interpreted with caution, and several confidence intervals, such as that for DCIS histology, are wide, reflecting this limited power. The association between PRR and re-excision was notable for appearing consistently across both the standard multivariable and the adaptive LASSO sensitivity analyses, which provides some reassurance against a chance finding; nonetheless, this result requires validation in a larger, adequately powered cohort before being considered robust.

Additionally, all three participating centers operate under the Swedish national standardized care pathway for breast cancer, which mandates a maximum of 35 calendar days from confirmed suspicion to the start of first treatment, with imaging typically performed within the first 1–2 weeks of this window; such short intervals make it unlikely that clinically meaningful volumetric tumor growth in most invasive cancers meaningfully confounded the observed PRR values [55, 56].

The trial protocol did not define strict indications for preoperative MRI and surgical approach. However, whilst this may introduce confounding related to institutional or surgeon-specific variability, it simultaneously reflects real-world practice in line with trial pragmatism. Furthermore, the median PRR of 1.25, denoting systematic underestimation of tumor size on imaging, appears difficult to reconcile with the low observed re-excision rate. This is explained by the trial’s fixed-margin protocol: because ORV was calculated by adding a standardized 1-cm margin to the imaging-based tumor extent, the median 25% underestimation observed in this cohort of predominantly small lesions (median 10.3 mm) corresponded to only 2–3 mm of additional tumor extent, well within this pre-specified margin. Re-excision was therefore required only in the minority of cases where discordance, often compounded by unfavorable tumor biology, exceeded what the standard margin could compensate for.

While causality cannot be inferred, the findings identify factors influencing re-excision and, more importantly, inform strategies for tailored precision surgery. Future studies should develop and validate biologically informed imaging–pathology prediction models that can reliably identify patients in whom radiological size truly reflects pathological extent, thereby enabling safe treatment de‑escalation and supporting the broader evaluation of minimally invasive approaches such as vacuum‑assisted excision in the SMALL trial and cryoablation in ICE3, whose success ultimately depends on accurate preoperative definition of the target volume [57, 58].

## Conclusion

Reoperation rates in BCS for non-palpable breast cancer are mainly driven by mismatches between radiologic and pathologic size and tumor biology. Although MRI improved tumor characterization in ILC, it did not reliably reduce reoperations or improve accuracy, and its tendency to overestimate size underscores the need to define target volume carefully to balance safety and cosmesis. As histology remains the key determinant of tumor extent, future strategies should integrate imaging with biological factors to guide surgical planning. Standardized MRI protocols, cost-effectiveness analyses, and studies on treatment de-escalation are needed to refine breast cancer management.

## Data Availability

No datasets were generated or analysed during the current study.
